# Recovery‐oriented nursing care based on cultural sensitivity in community psychiatric nursing

**DOI:** 10.1111/inm.12822

**Published:** 2020-12-06

**Authors:** Sumiko Matsuoka

**Affiliations:** ^1^ Department of Nursing Konan Women’s University Kobe Japan

**Keywords:** cultural sensitivity, nursing care, psychiatric nursing, qualitative research, recovery‐oriented care

## Abstract

Transforming to recovery‐oriented care is an urgent issue in community psychiatric nursing in Japan. Because traditional psychiatry is still influential, nurses are required to possess cultural sensitivity to objectively view conflicts between values when providing recovery‐oriented care. If recovery‐oriented care based on cultural sensitivity is clarified, it would help nurses providing recovery‐oriented care in non‐recovery‐oriented environments. Therefore, this study aimed to clarify recovery‐oriented nursing care based on cultural sensitivity in community psychiatric nursing in Japan. A semi‐structured interview with 21 community psychiatric nurses and participant observations for seven of them were performed. A qualitative description was undertaken to analyse the data. The relationships between categories were examined. The study conforms to the COREQ checklist. Through the analysis, six categories were revealed: 1. Continuously reflecting on one’s own practice and the influence of the traditional mental health culture; 2. Constructing a partnership with clients to uphold their rights and responsibilities; 3. Having client‐centred dialogue to help them enjoy life and grow; 4. Supporting clients’ lives and strengthening their self‐management; 5. Working as a team to achieve clients’ wishes, which includes some risks, and 6. Maintaining a relationship between clients and the people who care for them. Category 1 was central and enclosed by categories 2, 3 and 4. Categories 5 and 6 were located outside of categories 1 to 4. The results showed cultural sensitivity enables recovery‐oriented care even in non‐recovery‐oriented environments and include recognizing the traditional mental health culture, understanding clients’ experiences and accepting other’s values.

## Introduction

The dominant trend in mental health around the world is shifting from hospital‐based to community‐based care. As part of this trend, personal recovery has emerged, which refers to living a meaningful life with hope in spite of having restrictions caused by diseases (Deegan 1988) Personal recovery is different from clinical recovery, which refers to remission of symptoms or cure of mental illness, and emphasizes the centrality of hope, identity, meaning and personal responsibility (Davidson & Roe 2007; Slade 2009). In many countries, personal recovery has become a prominent concept in mental health (Leamy *et al*. 2011; van Weeghel *et al*. 2019) and recovery‐oriented care delivery has been promoted by national leadership (Department of Health 2001; U.S. President’s New Freedom Commission on Mental Health 2003; Australian Health Ministers 2003; New Zealand Mental Health Commission 2007; Mental Health Commission of Canada 2009).

Following the lead of other countries, Japan released ‘The Vision for Reform of Mental Health and Welfare’ to promote the transition from hospital‐based care to community‐based care in 2004 (Headquarters for Mental Health and Welfare of Ministry of Health, Labour and Welfare). However, more than 80% of psychiatric hospitals are private, making it difficult to decrease beds quickly (Kanata 2016), and the number of psychiatric care beds for the population has only fallen slightly since then and remains the highest in OECD countries (OECD 2014). Even in such a situation, social resources to support people with mental problems living in the community have increased gradually, including psychiatric home‐visit nursing. Since the number of psychiatric nurses working in the community has increased, converting to recovery‐oriented psychiatric nursing that is suited to community care from traditional psychiatric nursing which had been developed mainly in the hospital setting has become an urgent issue.

Although the concept of personal recovery has spread in mental health, providing recovery‐oriented care is not easy even in the community. The values of traditional psychiatry are still influential in the structure of the Japanese mental health system, which depends heavily on hospital‐based care. Nurses often work with care providers who do not prioritize personal recovery. Regardless of this, some community psychiatric nurses (CPNs) have started to provide recovery‐oriented care by focusing on clients’ hope and strengths, fostering their self‐determination and supporting fulfilment of their meaningful life. These CPNs should have the ability to see conflicts between values objectively and determine how to provide recovery‐oriented care in complicated situations. The ability to recognize one’s own values and those of others in an objective manner is referred to as cultural sensitivity (Suh 2004; Mahoney et al., 2006; Jirwe *et al*. 2009; Dudas 2012).

If recovery‐oriented care based on cultural sensitivity is clarified, it would help nurses who transition from psychiatric hospital care to community care provide recovery‐oriented nursing care. It might be also useful for CPNs who work with other professionals who do not share the value of recovery‐oriented care understand how it could be possible to provide recovery‐oriented nursing care.

## Background

A previous study on community psychiatric nursing clarified that cultural sensitivity is necessary for CPNs to recognize their own preconceptions about clients and their care that focuses on clients’ problems in order to form a positive attitude to support clients (Katakura *et al*. 2010). It was also clarified that the competency to work within an interdisciplinary team is essential for CPNs to provide recovery‐oriented care for clients (Walsh *et al*. 2012).

Some studies on recovery competencies for mental health professionals have shown the importance of: respecting clients’ values (Eriksen *et al*. 2013; Lakeman 2010), building a therapeutic alliance (Davidson *et al*. 2017; Femdal 2018; Khoury 2019; Ness *et al*. 2014; Russinova *et al*. 2013); supporting clients to have knowledge and skills to cope with mental illness (Lakeman 2010; Russinova *et al*. 2011); listening to clients’ narratives without making judgments based on their own values (Eriksen *et al*. 2013; Russinova *et al*. 2011; Williams & Tufford 2012); becoming aware of the stigma experienced by clients (Stuber *et al*. 2014); and recognizing clients’ trauma experiences in psychiatry (Lakeman 2010). It was also clarified that the workplace recovery culture was one of the predictors of recovery‐oriented competencies of professionals (Stuber *et al*. 2014).

As described above, some aspects of cultural sensitivity involving recovery‐oriented care have been illustrated in previous studies, but no previous studies have focused on cultural sensitivity among CPNs nor clarified the whole picture of recovery‐oriented nursing care based on cultural sensitivity. It would be useful to clarify how CPNs successfully perform recovery‐oriented nursing care based on cultural sensitivity in Japan.

Therefore, this study aimed to clarify recovery‐oriented nursing care based on cultural sensitivity in community psychiatric nursing in Japan. This study represents the first phase of a continuous study aiming to develop a model of recovery‐oriented nursing care based on cultural sensitivity in community psychiatric nursing.

## Methods

### Definitions

Key terms used in this study are defined as follows:

Recovery‐oriented care: Supporting people with mental health issues to live a meaningful life by focusing on clients’ hopes and strengths and fostering their self‐determination (Australian Health Ministers’ Advisory Council 2013; Mental Health Commission of Canada 2015; McKenna et al., 2016).

Culture: All activities of a group of people who live or work together. It includes the way of thinking, the way of seeing things, the way of relating to others, and behaviours that are learned and acquired within the group (Kleinman 1981; Spradley 1979).

Cultural sensitivity: Respecting others’ values, beliefs and ideas with recognition of one’s own values, beliefs and ideas. This also includes being aware of the values of mental health professionals and the position of power of nurses and other health professionals in their relationships with clients (Suh 2004; Kiefer 2006; Mahoney et al., 2006; Jirwe *et al*. 2009; Dudas 2012).

### Design

Qualitative description was used for this study. The qualitative description approach lies within the naturalistic approach, which creates an understanding of a phenomenon through accessing the meanings participants ascribe to them (Bradshaw *et al*. 2017), and it is relevant where information is required directly from those experiencing the phenomenon under investigation (Bradshaw *et al*. 2017; Neergaard *et al*. 2009). Qualitative description aims to produce a straight description and comprehensive summary of a targeted phenomenon using participants’ language and staying close to the data gathered (Kim *et al*. 2017; Sandelowski 2000, 2010). This study aimed to obtain straight descriptions about how skilled CPNs offered recovery‐oriented care based on cultural sensitivity. Therefore, qualitative description was thought appropriate for this study.

This study was carried out in two prefectures in the Kanto region, which includes Tokyo, and four prefectures in the Kansai region, which includes Osaka, in Japan. Home visits for people with mental illness are conducted mainly by psychiatric hospitals and visiting nursing stations in Japan. The number of assertive community treatment facilities (ACTs) is limited, but they provide impactful recovery‐oriented care. To maximize variation, CPNs belonging to the visiting section of psychiatric hospitals, visiting nursing stations and ACTs were recruited.

The Consolidated criteria for reporting qualitative research (COREQ) checklist (Tong *et al*. 2007) were applied as the reporting guideline for this study.

### Participants

Purposeful sampling was used to recruit CPNs. Visiting nursing stations, home visiting sections of psychiatric hospitals, and ACTs that had established the principle of recovery‐oriented practice were selected. The nurse managers of 10 workplaces were explained the purpose of this study and asked to recommend nursing staff that they recognized as excellent practitioners who provided recovery‐oriented care to clients. Inclusion criteria were as follows: (i) having at least 5 years of experience in community psychiatric nursing; and (ii) manager’s recommendation as an excellent practitioner who understands recovery‐oriented care well. Twenty‐one nurses were recommended by their managers. Each nurse received an explanation of the study and was informed that their participation was voluntary and that their anonymity would be protected. Written informed consent was obtained from all participants.

### Data collection

Semi‐structured interviews and participant observations of CPNs were conducted from January to March 2015. The script for the semi‐structured interviews was developed through a literature review, and revised through a pilot study designed by the author and conducted with two CPNs who were not participants of the present study (pilot study results were not published). Twenty‐one participants were interviewed individually in a private room at their workplace after their clinical duties or during free time while on duty. Since the term ‘cultural sensitivity’ was thought unfamiliar to participants, they were asked the following items: how they offer recovery‐oriented care; what they think is important for offering recovery‐oriented care; their relationships with clients in recovery‐oriented care; how they respect clients’ values and hopes; significant episodes when providing care towards clients’ recovery; and changes in nurses’ own values and practice when shifting from psychiatric ward to community‐based care. Interviews were recorded with each CPN’s consent.

Seven of the 21 participants consented to participant observations. They were asked to recommend some of their clients who they had visited for more than 1 year and who they thought would accept the researcher’s presence during their home visit. After obtaining the consent of clients and managers of the workplace, participant observations were held by following the participants’ regular home visit. One CPN conducted six home visits and two CPNs conducted two home visits, for a total of 13 home visit observations. The researcher sat beside the CPN during home visits and observed how the CPN interacted with the client. Field notes were made after the visit. Each visit took about 30 min.

### Data analysis

Transcribed data from the interviews and the written observations of nursing visits were analysed by the qualitative description method. All data were read repeatedly and text expressing CPNs’ practice and recognition of recovery‐oriented nursing care based on cultural sensitivity was extracted and named in short sentence as first codes. Then, the first codes were categorized based on similarity and named based on their common meaning as second codes. This categorization was repeated until the final codes were generated. The final codes were called categories and the codes that comprised the categories were called subcategories. The relationships between categories were examined with consideration of the cultural sensitivity behind each category and how the categories influenced each other.

### Rigour

According to Lincoln and Guba (1985), the rigour of a study is enhanced by fostering credibility, transferability, dependability and confirmability. Since the researcher met all participants for the first time during this study, the researcher explained the study purpose and motive before the interview and tried to create an environment in which participants felt comfortable talking openly about their recognition and daily practice. During the participant observations, the researcher sat to the side of the CPN, maintained a neutral expression and tried to minimize the effects of social desirability bias on the interaction between CPNs and clients in order to collect reliable data.

Three participants checked the results of the analysis to ensure credibility. Three nurse researchers who specialized in psychiatric nursing and had experience in qualitative research verified the validity of the results through comparison with their prior practice and knowledge of previous research. The descriptions of CPNs’ narratives are presented in detail in the Results section to assure transferability. The process of analysis was recorded to ensure dependability. The process of analysis was supervised by expert nurse researchers to establish confirmability.

## Results

### Participant characteristics

Table 1 shows the participants’ backgrounds. The participants were 21 CPNs (eight male, 13 female) with a mean age of 45.5 years. Their mean length of experience in community psychiatric nursing was 8.8 years (range: 5–17 years) and their mean length of experience in the psychiatric ward was 9.7 years (range: 0–17 years). One participant had no experience working in a psychiatric ward, but had more than 20 years’ experience as a public health nurse. Five of the CPNs were working at the visiting nursing section of a psychiatric hospital, 11 were working at visiting nursing stations, and five were working in ACTs. The mean length of interviews was 56.3 min (range: 35‐76 min).

**Table 1:** Nurse participants

Nurse	Sex	Age	Work place	Career length (years)		
				Psychiatric	Psychiatric	Participant
				home visits	hospital	observation
CPN01	Male	50s	Psychiatric hospital	17	13	1 home visit
CPN02	Female	50s	Psychiatric hospital	8	7	
CPN03	Female	40s	Psychiatric hospital	10	13	
CPN04	Male	40s	Psychiatric hospital	7	9	1 home visit
CPN05	Female	40s	Psychiatric hospital	5	11	1 home visit
CPN06	Female	40s	Visiting nursing station	11	12	6 home visits
CPN07	Female	40s	Visiting nursing station	6	8	
CPN08	Female	30s	Visiting nursing station	6	6	
CPN09	Male	30s	Visiting nursing station	6	12	
CPN10	Female	40s	Visiting nursing station	5	11	
CPN11	Male	30s	Visiting nursing station	6	7	
CPN12	Male	50s	Visiting nursing station	16	17	
CPN13	Female	40s	Visiting nursing station	9	1	1 home visit
CPN14	Female	60s	Visiting nursing station	15	8	
CPN15	Female	50s	Visiting nursing station	11	3	
CPN16	Male	40s	Visiting nursing station	11	7	2 home visits
CPN17	Male	30s	Assertive community treatment	5	5	1 home visit
CPN18	Male	40s	Assertive community treatment	7	14	
CPN19	Female	40s	Assertive community treatment	10	10	
CPN20	Female	40s	Assertive community treatment	8	3	
CPN21	Female	50s	Assertive community treatment	6	0	

Participant observations were held for seven CPNs. Three of them belonged to the visiting nursing section of a psychiatric hospital, three belonged to visiting nursing stations, and one belonged to an ACT.

### Categories of recovery‐oriented nursing care based on cultural sensitivity

The analysis identified 29 subcategories and six categories (Table 2). Each category is explained below. Narratives of representative nurses are shown in italics.

**Table 2:** Categories and subcategories

Categories	Subcategories
1. Continuously reflecting on one’s own practice and the influence of the traditional mental health culture	Being aware of the influence of the mental health environment for nursing practice in the psychiatric ward Reflecting on nurses’ own practice of client‐centred care Being careful not to offer values Being careful not to offer recovery to clients
2. Constructing a partnership with clients to uphold their rights and responsibilities	Providing a sense of relief for clients based on an understanding of their prior experience in psychiatry Meeting clients as equal community residents to relinquish power as a healthcare professional Supporting clients in maintaining a stable condition through a good relationship Believing that clients have power and sharing their strengths and goals with them Respecting clients’ values to meet their needs Respecting clients’ rights, including the right to fail, and supporting their decision making
3. Having client‐centred dialogue to help them enjoy life and grow	Having dialogue that helps clients feel relief based on an understanding of their prior experience in psychiatry Enjoying dialogue with humour, which brings brightness to clients’ lives Listening to clients’ narratives without judgement to understand them Clarifying clients’ hopes through the dialogue Listening to clients’ narratives about their illness experience to support them in finding another meaning of having an illness
4. Supporting clients’ lives and strengthening self‐management	Trying to understand problematic client behaviours or symptoms as natural human reactions Confirming both the influence of symptoms on clients’ lives and their strengths Collaborating with clients to cope with difficulties to make their lives more comfortable Collaborating with clients in various ways to improve or maintain their health in addition to medication based on the understanding that prioritizing medication can destroy the relationship with the client Collaborating with clients about medication compliance and appropriate dose based on the understanding that it is difficult to continue medication Viewing clients’ illness and treatment as only one part of their lives
5. Working as a team to achieve clients’ wishes, which includes some risks	Supporting clients’ hopes by focusing on progress even if it includes some risks or looks difficult to achieve Sharing the policy of supporting clients’ hopes with team members Sharing the responsibility of decision making through discussion in a non‐hierarchical team Accepting clients’ hopes as much as possible with frequent discussion with team members when clients’ condition declines
6. Maintaining a relationship between clients and the people who care for them	Supporting clients in using social resources that they need and building interpersonal relationships Constructing a network with people in the community or in other institutions who care for clients Supporting clients’ families and mediating the relationship between clients and their families Listening without taking initiative to solve clients’ problems

#### Category 1: Continuously reflecting on one’s own practice and the influence of the traditional mental health culture

The participants unanimously referred to the necessity of continuous reflection on their own care based on the recognition that they were easily influenced by the traditional mental health culture which had been created through the values of a biomedical approach, professionals’ role in the workplace, the construction of the mental health system, and society’s expectations of mental health professionals. CPNs 09, 16 and 19 stated that they noticed the care they provided in the psychiatric ward was not applicable in the community through clients’ rejection, and looked back on psychiatric ward care that was customarily governed by regulations and implied rules. CPNs 06, 08, 14 and 21 stated the importance of continuing to reflect on their daily care in the community and whether it was client‐centred. CPN16 noted the importance of noticing nurses’ tendency towards administrative tasks and its background through reflection as follows:

When nursing a user at home, you cannot apply the same ways of thinking or do things the same way as in a hospital ward. That may cause problems and the relationship between the user and nurses will be strained. For example, clients could refuse to take medication. In cases like these, you worry about what to do. If you look back to what you did in the ward, because ward nursing is your foundation, you notice that you ended up forcing things. In the hospital, a patient will not be discharged if they do not take their meds. The relationship unavoidably becomes hierarchical, and the patient has to comply. After all, the ward is locked.

##### Category 2: Constructing a partnership with clients to uphold their rights and responsibilities

This category clarified that CPNs built a relationship with clients as equals to support their making decisions and taking on responsibilities. This was based on the understanding that clients had experienced an unequal relationship with nurses in psychiatric wards and saw nurses as being in a position of power. Therefore, all participants stated that they were thoughtful of relinquishing their power as nurses and empowering clients. In observation of home visits, CPNs 01 and 04 behaved politely as a guest, which allowed the client to act as a host. Then, they respected clients’ ways of living. Furthermore, the importance of respecting clients’ decisions, including their right to fail, was stated by many participants (CPNs 06, 08, 09, 12, 18, 21). CPN21 thought that overprotection was a violation of human rights, and she indicated how she respected clients’ rights as follows:

Most important is understanding that if the user is a 30‐year‐old woman with a disability, for example, they are naturally able to experience and feel the same things experienced and felt by a 30‐year‐old woman without a disability. They also have a right to make mistakes. How do you support them in that? It is better to avoid unnecessary mistakes, so of course you prepare to do that. However, being more protective than necessary is not conducive to recovery and may interfere with the person’s rights.

##### Category 3: Having client‐centred dialogue to help them enjoy life and grow

This category clarified that having dialogue and listening to clients supported them to in enjoying life, creating hope for the future and finding the meaning of their illness experience. This was based on understanding of clients’ experiences with professional‐initiative dialogue in the psychiatric ward, in which clients were asked about their symptoms and told what they should do. At the first, participants (CPNs 01, 06, 11, 14, 15) stated the importance of enjoying dialogue with humour, which brought brightness to the lives of clients with challenging symptoms. The importance of maintaining the attitude of not judging or blocking clients’ narrative was emphasized by CPNs 04, 09 and 12. It was thought that this attitude made the clients feel safe to talk and express their hopes through dialogue. During the observation of home visit, CPN17 listened to the client who was working as a peer supporter talk about his recovery journey by overcoming the distress of illness. CPN17 highlighted the importance of supporting clients to find alternative meanings of having an illness through dialogue as follows:

Encourage the idea that the experience of illness or having had an illness is meaningful for the person. When talking to someone in a state of mind where they say things like, “I have no hope, nothing. There is no god,” you can say something like, “That experience, what did it mean to you?” I feel that finding new meaning is probably an important first step for that person to have hope. That is very hard for a person to do alone. It is also hard to do with someone who is very familiar with the user’s circumstances. Someone who does not really know much about these circumstances but has an interest in the person can listen and discuss what meaning the events hold for them and in doing so help them create a new alternative story.

##### Category 4: Supporting clients’ lives and strengthening their self‐management

This category clarified that CPNs were released from thinking that follows medical model and focused on clients’ strengths and supported clients to improve their lives in various ways, besides just medication. This was based on the value of viewing a client not as a person with mental disease but as a person who had serious distress that prevents them from living a full life. What was noted by many participants was the importance of the attitude of not managing client medication, because they recognized that putting priority on medication destroyed their relationship with clients. Instead, they tried to improve clients’ lives because they thought that it brought them mental stability. In the observation of home visits, CPNs 04 and 06 thought together with clients to find various ways of making their lives more comfortable. CPN16 indicated how he worked together with clients to improve their self‐management as follows:

I ask them frankly whether or not they have taken their meds, and if the user says “I have not,” I say something like, “Oh really. OK. How do you feel?” Or something like, “Really? Well what should we do?” It is okay if their mental state and illness appeared to be under control, even though they were not taking their medications. (some text omitted) If they bring up a subject like living a better regulated life, you can say something like, “Well, what do you think you can do to accomplish that?” You can talk about how they can manage their own health. In short, this is because it is okay if they can continue to live in their community.

##### Category 5: Working as a team to achieve clients’ wishes, which includes some risks

This category clarified that CPNs supported clients’ wishes by examining the various opinions of team members in spite of the risks or difficulties involved. Although participants understood that respecting clients’ hopes was essential for personal recovery, they felt it was difficult to decide whether they would support clients’ challenges because of the responsibility to protect clients from serious disadvantage. So CPNs 06, 14, 17, 18 and 19 insisted on the importance of discussion with team members to share responsibility for the decision. If the client’s condition worsened, it would become more difficult to respect the client’s wishes. CPN19 pointed out that more frequent discussions were needed to achieve impartial assessment of the client’s condition and respect his/her wishes as much as possible. CPN18 emphasized the effectiveness of discussion in non‐hierarchical teams to reach appropriate decisions for supporting clients’ wishes as follows:

If the user has an interest in something, I try to accommodate it to the maximum extent possible if the user wishes, even if it would be inconceivable at the hospital. From this point onward, there is no saying “no.” If something must be done, you do it. For example, a user said, “I would like to once more visit a place that has memories for me. My father took me there when I was still around elementary school age.” We talked about how to save up the money needed, and when enough was set aside, we went. If it is beneficial for the user and approved by everyone on the team, it is okay. The relationships among team members are equal, so information is shared quickly, and anything can be discussed.

##### Category 6: Maintaining a relationship between clients and the people who care for them

This category clarified that CPNs constructed relationships with other professionals and non‐professionals who care for clients and listened with an impartial attitude. It was noted that it is important to support clients’ families and mediate the relationship between clients and their families during regular home visits by CPNs 01, 06 and 07. During home visit observations, CPNs 01 and 06 took time to listen to clients’ family members. In addition, many participants (CPNs 06, 07, 12, 14, 15) stated that they supported clients in using social resources and building interpersonal relationships with people other than CPNs. They also tried to make contact with people who care for clients to create a support network. However, the network of people who care for clients became more important when problems concerning clients occurred. CPN17 indicated having the attitude that he did not take the initiative to solve a problem and instead listened to the opinions of those involved to reach a mutually acceptable compromise as follows:

I think it is important to be the person who knows the most about the circumstances of the user, the other people in the community, and the user’s family. The needs to be considered are those of the people in charge of public assistance, real estate agents (renting an apartment), and the user. I think the only choice in that case is to find a balance between these needs. At that point, the needs of everyone involved should be properly and fully listened to. When I go and listen to what everyone has to say, someone may say something like, “This is how I can do it.” The circumstances may improve to the point that everyone can compromise.

### Relationships between categories

Relationships between the six categories are as follows. [1. Continuously reflecting on one’s own practice and the influence of the traditional mental health culture] was placed in the centre as fundamental to recovery‐oriented nursing care based on cultural sensitivity and supported [2. Constructing a partnership with clients to uphold their rights and responsibilities], [3. Having client‐centred dialogue to help them enjoy life and grow], and [4. Supporting clients’ lives and strengthening their self‐management]. Practice based on understanding clients’ experiences facilitated CPNs’ reflection on their own practice; therefore, it was thought that categories 2, 3 and 4 facilitated category 1.

Building a partnership facilitated client‐centred dialogue and strengthened clients’ self‐management. Client‐centred dialogue also helped build the partnership and strengthen clients’ self‐management. Strengthening clients’ self‐management without offering one’s own beliefs of the medical model helped build the partnership and further client‐centred dialogue. Therefore, it was thought that categories 2, 3 and 4 facilitated each other. Categories 1 to 4 were thought to be nursing care between clients and CPNs based on the cultural sensitivity of recognizing the traditional mental health culture and understanding clients’ experiences.

[5. Working as a team to achieve clients’ wishes, which includes some risks] and [6. Maintaining a relationship between clients and the people who care for them] were thought of as care practised in collaboration with the many people who care for clients. These categories were based on the care practised between clients and CPNs and also represented positive circumstances in recovery‐oriented nursing care between clients and CPNs. Therefore, it was thought that these two categories and categories 1 to 4 influenced each other.

Working as a team to support clients’ wishes was the foundation for collaboration with other people who were outside of the support team, which helped the team take risks for clients’ recovery. Therefore, it was thought that categories 5 and 6 facilitated each other. These two categories were thought to be nursing care practised in collaboration with the many people who care for clients and were based on the cultural sensitivity of accepting various opinions.

**Fig. 1:**
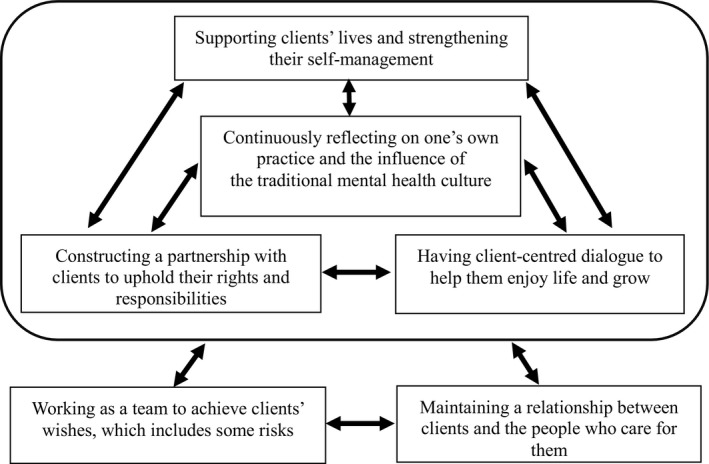
Categories of recovery‐oriented nursing care based on cultural sensitivity in community psychiatric nursing and the relationships between them. Black arrows indicate that the categories influence each other.

The overall relationships between the six categories are presented in Figure 1.

## Discussion

In recovery‐oriented nursing care based on cultural sensitivity, CPNs’ reflection on their own practice and the influence of the traditional mental health culture were placed in the centre. The importance of CPNs’ cultural sensitivity to be aware that they were influenced by their professional education, the professionals’ role in the workplace, the construction of the mental health system, and society’s expectations of mental health professionals was clarified. Japan has still many psychiatric care beds and depends on hospital‐based care in spite of policies aimed towards community‐based care. Moreover, the concept of personal recovery has spread but it has not become national policy yet. In addition, the stigma of mental illness and long‐term hospitalization policies that have continued for a long time still exist in Japanese society and inhibit deinstitutionalization (Kanata 2016). To provide recovery‐oriented care in a non‐recovery environment like Japan, it was essential for CPNs not to focus on their own recognition and practice, but rather to broaden their views about the context of psychiatry.

Perceiving the context of psychiatry promoted deep understanding of clients’ previous experience in psychiatry. There can be great difference between the points of view of medical professionals and clients (Kleinman 1988; Kiefer 2006), and this gap is remarkable in psychiatry, which often includes compulsory treatments, non‐voluntary admission and restraint (Allikmets *et al*. 2020; Staniszewska *et al*. 2019).

Focusing on the client’s subjective opinion was fundamental to recovery‐oriented care, so the cultural sensitivity of imagining the clients’ experience from their standpoint is crucial. In this study, CPNs noticed that clients had previously experienced unequal relationships and dialogue with nurses who were in a position of power and provided treatment and care based on the medical model, especially in the psychiatric ward. This acknowledgement helped CPNs relinquish their power and beliefs as professionals, and they found clients’ strengths and hopes, which are connected to their recovery. The importance of relinquishing professional power in recovery‐oriented care has already been indicated (Cutcliffe & Happell 2009; Walsh *et al*. 2008), but it becomes more important for CPNs to provide recovery‐oriented care in environments that still depend on hospital‐based psychiatry.

Furthermore, a third cultural sensitivity of accepting the various values of other care providers to support the client together was clarified. It was especially urgent in difficult situations such as when clients hoped to overcome a challenge, including risks, or when problems concerning the client came up. To support clients, taking risks that make them grow or improve is essential for recovery‐oriented care (Felton & Stacey 2008; Holley *et al*. 2016). However, it is not easy to decide whether to support clients’ challenges or not. CPNs had frequent discussions with colleagues and accepted various opinions to achieve impartial assessment of clients’ condition and the level of risk for offering recovery‐oriented care.

When problems concerning the client came up, CPNs did not take the initiative to solve the problems and tried to hear the opinions of the people concerned and waited until they could reach common ground. This suggested that CPNs could deal with the conflict among various values.

The main limitation of this study is that the results were influenced by the circumstances of psychiatry and culture in Japan. However, this study is expected to be useful for CPNs in other countries who want to offer recovery‐oriented care in environments where the value of recovery‐oriented care is not sufficiently shared among care providers. Another limitation of this study is that participant observations were held for only seven CPNs. If more observations had been held, richer data would have been collected.

In spite of the limitations of this study, the results for recovery‐oriented care based on cultural sensitivity were thought useful for CPNs who are working in non‐recovery environments. It is expected that a model of recovery‐oriented nursing care based on cultural sensitivity in community psychiatric nursing will be developed.

## Conclusion

This study clarified six categories of recovery‐oriented nursing care based on cultural sensitivity and the relationships between them. The results showed that cultural sensitivity enables recovery‐oriented care even in environments where the values of recovery‐oriented care are not shared enough, and recognizing the traditional mental health culture, understanding clients’ experiences and accepting various values of others is important.

## Relevance for clinical practice

The results of this study can provide useful information for CPNs transitioning to community‐based care to help them understand and practice recovery‐oriented care for clients. This study shows how CPNs deliver recovery‐oriented care in various situations in non‐recovery‐oriented settings by using their cultural sensitivity. Providing recovery‐oriented care becomes difficult when CPNs are unconscious of their own values, the influence of the traditional mental health culture, nurses’ position of power, and their insufficient ability to accept various values of people who care for clients.

## Funding

This study was a part of a research project funded by Grants in Aid for Scientific Research in Japan from 2012‐2016 (no. 26463515).

## Ethical considerations

Each participant received an explanation of the study and was informed that their participation was voluntary and that their anonymity would be protected. Written informed consent was obtained from all participants. This study was approved by the research ethics committee of Kobe City College of Nursing (Data of approval: 19 February 2014, Approval number; 2014‐2‐19).
